# Bacterial, Antimicrobial, and Clinical Profile of Multidrug-Resistant Gram-Negative Bacilli Isolates From Intensive Care Unit Patients

**DOI:** 10.7759/cureus.109062

**Published:** 2026-05-17

**Authors:** Vaishnavi G Chavan, Satyajeet Pawar, Satish Patil

**Affiliations:** 1 Department of Microbiology, Krishna Institute of Medical Sciences, Krishna Vishwa Vidyapeeth (Deemed to Be University), Karad, IND

**Keywords:** antibiotic susceptibility, antimicrobial resistance, healthcare-associated infections, intensive care unit, multidrug-resistant gram-negative bacilli

## Abstract

Background

Multidrug-resistant (MDR) Gram-negative bacilli (GNB) are a major cause of healthcare-associated infections in intensive care units (ICUs), leading to increased morbidity, mortality, and limited treatment options. This study was conducted to determine the prevalence of MDR-GNB among ICU clinical specimens and to evaluate their antimicrobial susceptibility patterns.

Material and methods

This cross-sectional observational study was carried out at the Department of Microbiology, Krishna Institute of Medical Sciences, Karad, from August 2024 to March 2025. A total of 370 clinical specimens obtained from ICU patients were processed using standard microbiological methods. The VITEK 2 compact automated system (Marcy-l'Étoile, France: bioMérieux) was used to identify Gram-negative bacilli, and VITEK 2 AST cards were used for antibiotic susceptibility testing. Multidrug-resistant isolates were defined as resistance to three or more antimicrobial classes.

Results

A total of 370 specimens were processed; growth was observed in 228 specimens. Among these, 109 were Gram-positive, and 119 were Gram-negative. Multidrug-resistant Gram-negative bacteria were identified in 101 isolates. The highest burden of infection was observed in both the 31-40 year and 41-50 year age groups, with each group contributing 26 cases (25.74%). Males were more frequently affected than females, contributing 69 (68.32%) cases compared to 32 (31.68%). Among the pathogens, *Pseudomonas aeruginosa *was the most commonly isolated organism (31, 30.69%), followed by Klebsiella spp. (25, 24.75%), and *Klebsiella pneumoniae *(18, 17.82%). *Escherichia coli *(12, 11.88%) and Acinetobacter spp. (11, 10.89%), were also notable contributors. Urine was the predominant specimen source (43, 42.57%), followed by endotracheal tube samples (22, 21.78%), wound swabs (10, 9.90%), sputum (9, 8.91%), and blood (9, 8.91%). Antimicrobial resistance was high for meropenem (96, 95.05%), netilmicin (94, 93.07%), amikacin (93, 92.08%), and ceftazidime (92, 91.09%). In contrast, nitrofurantoin showed the lowest resistance (5, 11.63%) and the highest susceptibility (38, 88.37%) among urine isolates. The second most effective antimicrobial combination was piperacillin/tazobactam, with susceptibility observed in 34 (33.66%) isolates. These findings indicate a significant prevalence of MDR-GNB infections among middle-aged male intensive care unit (ICU) patients, with limited effective treatment options.

Conclusion

This study demonstrates a high prevalence of multidrug-resistant Gram-negative bacilli among patients in the intensive care unit with *Pseudomonas aeruginosa*, Klebsiella spp., Acinetobacter spp., and *Escherichia coli* as the predominant pathogens. The predominance of urine and respiratory specimens may be associated with the frequent use of urinary catheters and mechanical ventilation in critically ill ICU patients, contributing to hospital-acquired infections. The high level of resistance across multiple antimicrobial classes, including carbapenems, aminoglycosides, cephalosporins, fluoroquinolones, and piperacillin/tazobactam, highlights the challenge of having few options for treatment. These results show the urgent need for early microbiological diagnosis, culture, and susceptibility-guided therapy; strict methods for infection prevention and control; and robust antimicrobial stewardship programs to reduce the burden of MDR infections and improve patient outcomes in the ICU.

## Introduction

Multidrug-resistant organisms (MDROs) are defined as pathogens that exhibit resistance to at least one antimicrobial agent in three or more antimicrobial classes [1,2]. These organisms represent a serious and rapidly emerging threat to patient safety worldwide. Among them, multidrug-resistant (MDR) Gram-negative bacilli (GNB) have become a major global health concern, particularly in critical care settings such as intensive care units (ICUs), where patients are highly vulnerable to infections due to invasive procedures, prolonged hospital stays, and prior exposure to broad-spectrum antibiotics [3,4].

Infections caused by MDR-GNB are associated with increased morbidity, mortality, and healthcare costs [5]. Clinically significant GNB commonly encountered in ICUs include *Klebsiella pneumoniae, Escherichia coli, Pseudomonas aeruginosa, *and* Acinetobacter baumannii*. These pathogens are frequently implicated in bloodstream infections, ventilator-associated pneumonia, urinary tract infections, and wound infections [6,7]. They demonstrate increasing resistance to multiple antibiotic classes, including β-lactams, carbapenems, aminoglycosides, and fluoroquinolones, thereby significantly limiting therapeutic options. In such cases, last-resort antibiotics such as colistin and tigecycline are often required [8].

The emergence of antimicrobial resistance in Gram-negative bacilli is driven not only by extended-spectrum β-lactamases (ESBLs) and carbapenemase production but also by AmpC β-lactamases, which may be either chromosomal or plasmid-mediated. AmpC enzymes confer resistance to expanded-spectrum cephalosporins, cephamycins, and β-lactam/β-lactamase inhibitor combinations, further reducing available treatment options in ICU settings [9].

To reduce the burden of MDR infections in critically ill patients, it is essential to implement effective antimicrobial stewardship programs and maintain continuous surveillance of resistance patterns [1]. However, antibiotic susceptibility profiles of pathogenic bacteria vary significantly between hospitals and even among different departments within the same institution [10]. Therefore, the present study was conducted to determine the prevalence of MDR Gram-negative bacilli in ICU specimens and to provide evidence to guide empirical antibiotic therapy in critically ill patients.

## Materials and methods

Study design and sample size

The present cross-sectional observational study was conducted in the Department of Microbiology, Krishna Institute of Medical Sciences, Krishna Vishwa Vidyapeeth, and Krishna Hospital and Medical Research Centre, Karad, from August 2024 to March 2025. The sample size for this study was calculated based on a previously reported prevalence of 64% by Chaudhuri and Gupta [11]. Using this prevalence, the sample size was determined by applying the standard formula as follows: n = 4pq/L^2^. Here, p represents the prevalence (64%), q is calculated as (100-p) = 36, and L denotes the allowable error (precision).

n = 4pq/L^2^

= 4 × 64 × 36/10^2^

= 92

Thus, using the prevalence reported by Chaudhuri and Gupta, the minimum required sample size was calculated to be 92 [11]. During the study period, 370 clinical specimens were processed, of which 228 showed microbial growth. Among these, 119 were Gram-negative isolates, and 101 fulfilled the criteria for multidrug resistance; therefore, these 101 MDR isolates were included in the final analysis to strengthen the validity of the study findings.

Inclusion and exclusion criteria

Consecutive clinical isolates of multidrug-resistant Gram-negative bacilli obtained from clinical specimens of patients admitted to different intensive care units, including the medicine ICU, surgery ICU, neurology ICU, and pediatric intensive care unit (PICU), involving all age groups and both genders, were included in the present study. Gram-negative bacterial isolates resistant to two or fewer classes of antimicrobials, Gram-positive organisms, and duplicate isolates obtained from the same patient were excluded from the analysis.

Specimen collection and processing

After obtaining approval from the Institutional Ethics Committee of Krishna Vishwa Vidyapeeth (Deemed to be University) (#KVV/IEC/05/2024), a total of 370 clinical samples from ICU-admitted patients at Krishna Hospital were processed during the study period. Informed written consent was obtained from all study participants. Among the processed specimens, 101 isolates were identified as multidrug-resistant Gram-negative bacilli. Clinical specimens, including urine, sputum, pus, blood, body fluids, endotracheal tube secretions, and other relevant ICU samples, were collected aseptically from patients of all age groups. All specimens were collected aseptically in sterile containers and processed according to standard microbiological protocols. A direct smear was prepared from each sample and stained using the Gram stain method for preliminary evaluation. Microscopic examination under oil immersion was performed to detect Gram-negative bacilli. Morphological characteristics, including size, shape, and arrangement of the organisms, were carefully observed and recorded for further identification.

Isolation and Identification of Bacterial Isolates

All clinical specimens were cultured on MacConkey agar, blood agar, and chocolate agar, followed by incubation at 37°C for 18-24 h under aerobic conditions. The isolated colonies were initially subjected to Gram staining for preliminary identification. For species-level identification, Gram-negative bacilli were analyzed using the VITEK 2 compact automated system with GN 21341 identification cards (Marcy-l'Étoile, France: bioMérieux).

Antimicrobial Susceptibility Testing

Antimicrobial susceptibility testing was performed using the VITEK 2 compact automated system with VITEK 2 antimicrobial sensitivity test (AST)-N405 and AST-N406 cards. The results were interpreted and categorized as susceptible, intermediate, or resistant.

Resistance Categorization (R1, R2, R3)

According to the antimicrobial susceptibility results, isolates were categorized based on the number of antimicrobial classes to which they exhibited resistance. R1 - isolates resistant to at least one antimicrobial agent in one class. R2 - isolates resistant to antimicrobial agents in two different classes. R3 - isolates resistant to at least three antimicrobial classes were classified as multidrug-resistant (MDR).

## Results

**Figure 1:**
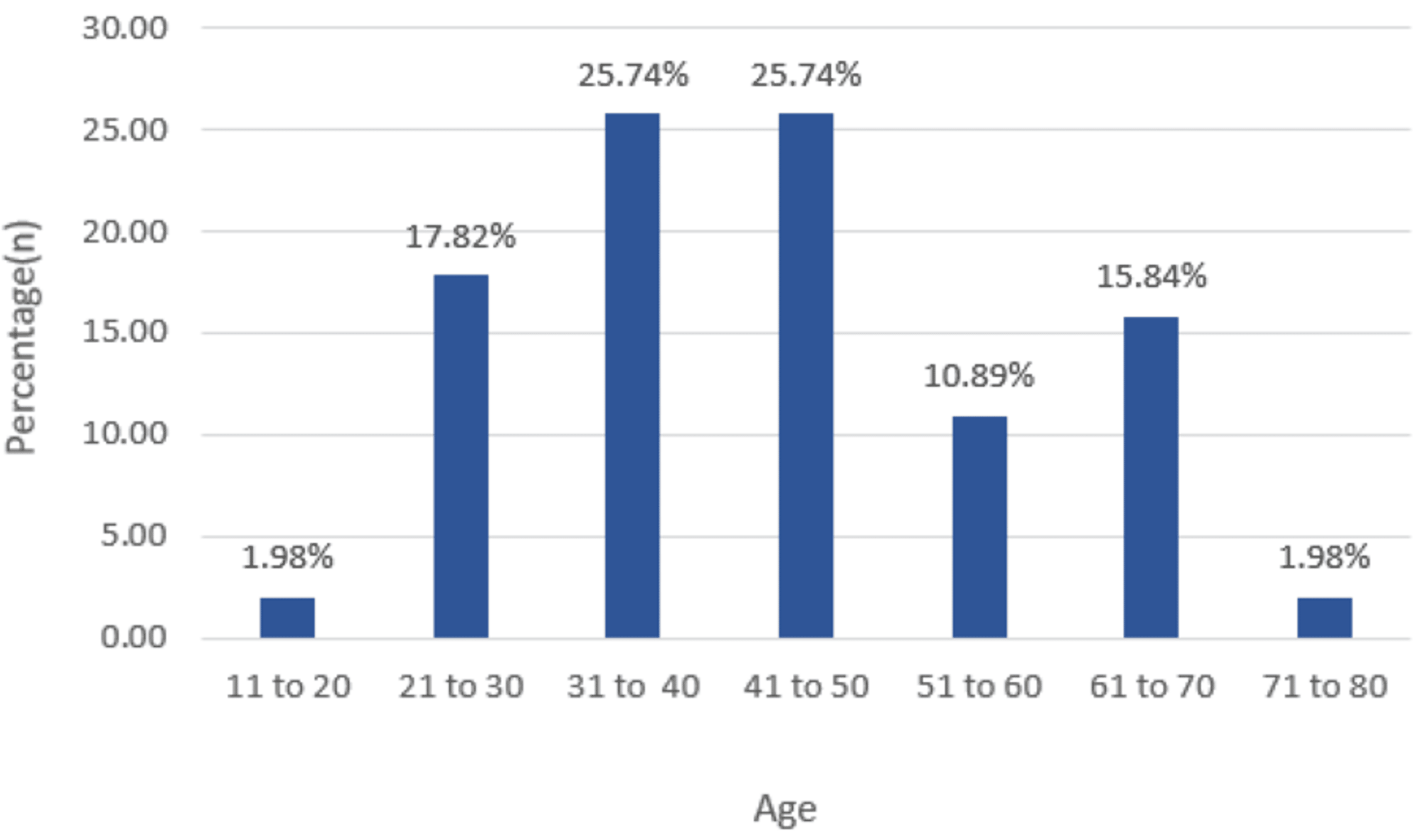
Age-wise distribution of multidrug-resistant Gram-negative bacilli from ICU. n: number

Among the 370 clinical specimens processed, 228 showed microbial growth. Of these, 109 were Gram-positive, and 119 were Gram-negative. Among the Gram-negative isolates, 101 were identified as multidrug-resistant Gram-negative bacilli (MDR-GNB). The age-wise distribution showed considerable variation in infection rates across age groups. The lowest prevalence was observed in the 11-20 years age group (2, 1.98%), while the highest prevalence was noted in the 31-40 years and 41-50 years age groups (26, 25.74% each). The 21-30 years age group accounted for 17.82% (18). A decline in prevalence was observed in older age groups, particularly among those aged 71-80 years (2, 1.98%). These findings indicate a peak in MDR-GNB infections among middle-aged individuals, with lower prevalence in younger and older age groups (Figure 1).

**Figure 2:**
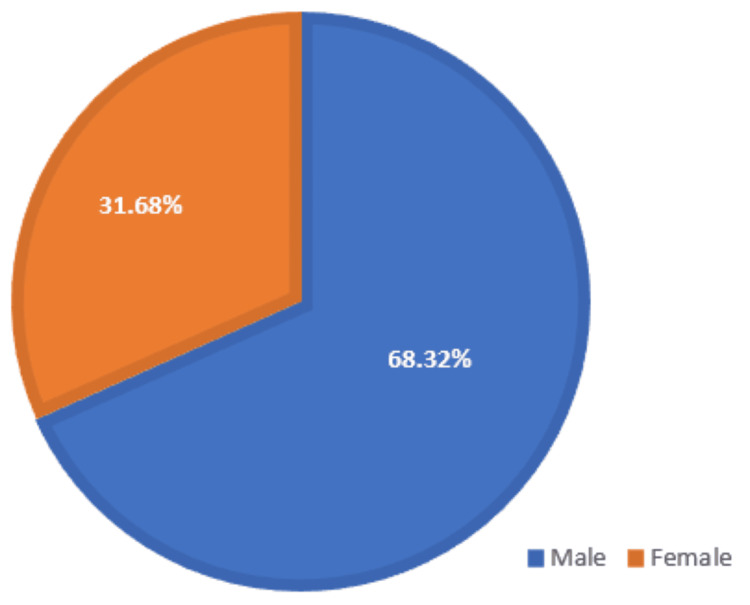
Gender-wise distribution of MDR Gram-negative bacilli among clinical isolates from ICU patients. MDR: multidrug-resistant

**Figure 3:**
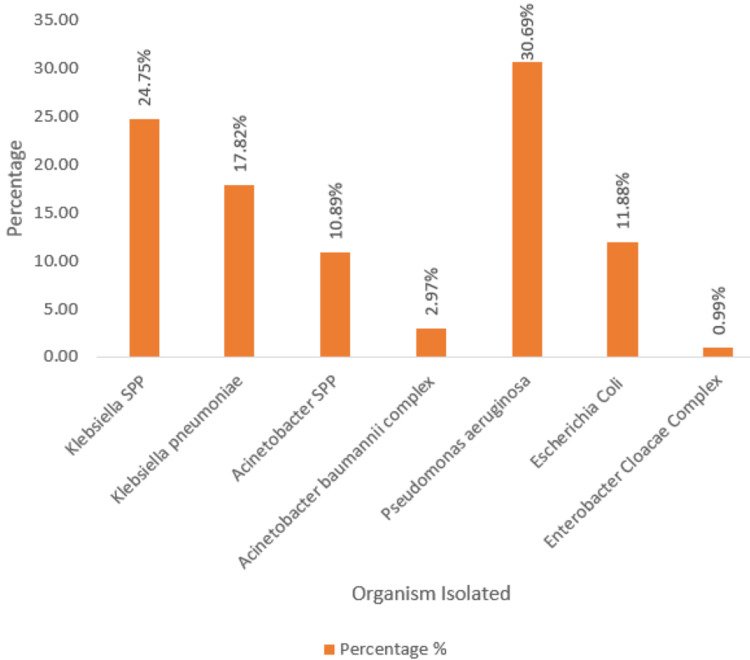
Organism-wise distribution of multidrug-resistant Gram-negative bacilli isolated from ICU patients. SPP: species

Among the 101 multidrug-resistant Gram-negative bacilli isolates obtained from intensive care unit (ICU) patients, a male predominance was observed, with 69 isolates (68.32%) from male patients and 32 isolates (31.68%) from female patients (Figure 2). Among the 101 multidrug-resistant Gram-negative bacilli isolates, *Pseudomonas aeruginosa* was the most frequently recovered organism, accounting for (31) 30.69%. This was followed byKlebsiella spp. (25, 24.75%), and *Klebsiella pneumoniae *(18, 17.82%). Other isolates included *Escherichia coli* at 11.88% (12), *Acinetobacter spp*. at 10.89% (11), and the *Acinetobacter baumannii *complex at 2.97% (3). The least frequently isolated organism was *Enterobacter cloacae *complex*,* with only 0.99% (1). Overall, non-fermenting Gram-negative bacilli, particularly *Pseudomonas aeruginosa*, predominated in ICU infections, followed by *Klebsiella species* (Figure 3).

Among the 119 Gram-negative isolates, 101 were identified as multidrug-resistant (MDR), while 18 were non-MDR isolates. Among the MDR isolates, *Pseudomonas aeruginosa* was the most commonly isolated organism (31, 30.69%), followed by Klebsiella spp. (25, 24.75%), *Klebsiella pneumoniae* (18, 17.82%), *Escherichia coli* (12, 11.88%), and Acinetobacter spp. (11, 10.89%) (Table 1).

**Table 1:** Distribution of multidrug-resistant Gram-negative bacteria in ICU clinical isolates. MDR: multidrug-resistant; RG3: resistant to three groups of antibiotics; RG4: resistance observed in four antimicrobial classes; RG5: resistance observed in five or more antimicrobial classes; spp: species

Bacterial isolates	RG3	RG4	RG5	MDR (RG3+ RG4+RG5), n (%)
Klebsiella spp.	6	7	12	25 (24.75)
Klebsiella pneumoniae	4	6	8	18 (17.82)
Acinetobacter spp.	4	2	5	11 (10.89)
*Acinetobacter baumannii *complex	0	2	1	3 (2.97)
Pseudomonas aeruginosa	7	10	14	31 (30.69)
*Escherichia coli*	4	2	6	12 (11.88)
*Enterobacter cloacae *complex	0	0	1	1 (0.99)
Total	25	28	48	101 (100)

A total of 101 multidrug-resistant Gram-negative bacilli isolates from patients in intensive care units were analyzed based on specimen type. The majority of isolates were recovered from urine specimens (43, 42.57%), followed by endotracheal tube (ETT) specimens (22, 21.78%). Other sources included pus and wound swabs (10, 9.90%), sputum (9, 8.91%), and blood (9, 8.91%). A smaller proportion of isolates were obtained from body fluid samples (5, 4.95%). Minimal isolates were obtained from central venous pressure (CVP) tips, tracheostomy tubes, and Foley catheter tips, each contributing 1 (0.99%). Overall, urine and respiratory samples (ETT and sputum) constituted the major sources of MDR Gram-negative bacilli in ICU patients (Table 2).

**Table 2:** Specimen-wise distribution of MDR Gram-negative bacilli. MDR: multidrug-resistant; ETT: endotracheal tube; CVP tip: central venous pressure catheter tip

Specimen type	n	%
Urine	43	42.57
ETT	22	21.78
Sputum	9	8.91
Pus/wound swab	10	9.90
Fluids	5	4.95
Blood	9	8.91
CVP tip	1	0.99
Tracheostomy tube	1	0.99
Tip of Foley catheter	1	0.99
Total	101	100

**Figure 4:**
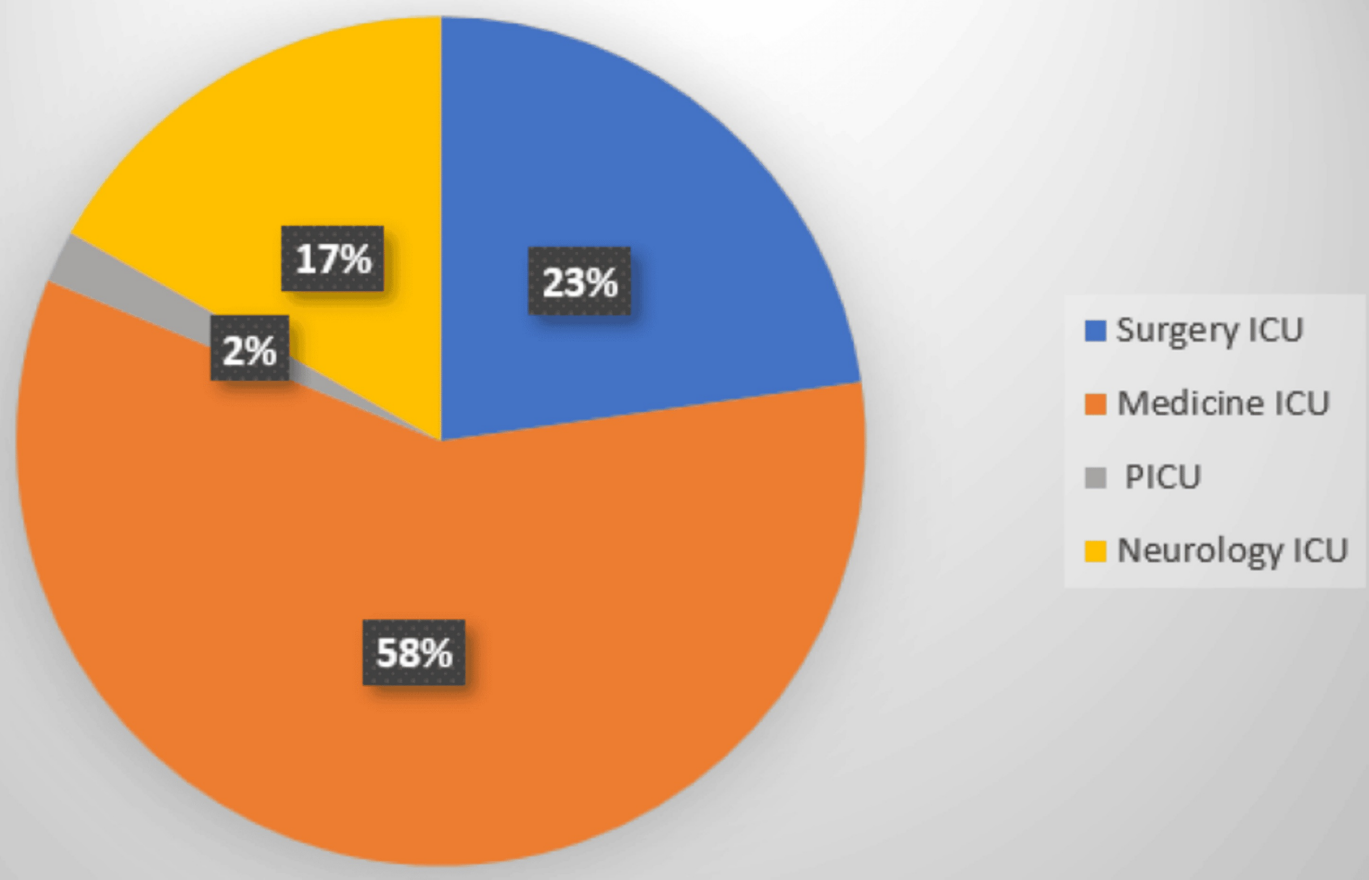
Department-wise distribution of MDR Gram-negative bacilli in ICU. Data in the figure are expressed as percentages rounded to the nearest whole number. MDR: multidrug-resistant; PICU: pediatric intensive care unit

A total of 101 multidrug-resistant Gram-negative bacilli (MDR-GNB) isolates were distributed across various intensive care unit (ICU) departments. The highest number of isolates was reported from the medicine ICU, contributing 59 isolates (58.42%), followed by the surgery ICU with 23 isolates (22.77%). The neurology ICU accounted for 17 isolates (16.83%), while the pediatric intensive care unit (PICU) contributed two isolates (1.98%). Overall, the burden of MDR Gram-negative infections was highest in the medicine ICU compared to other ICU settings (Figure 4).

**Figure 5:**
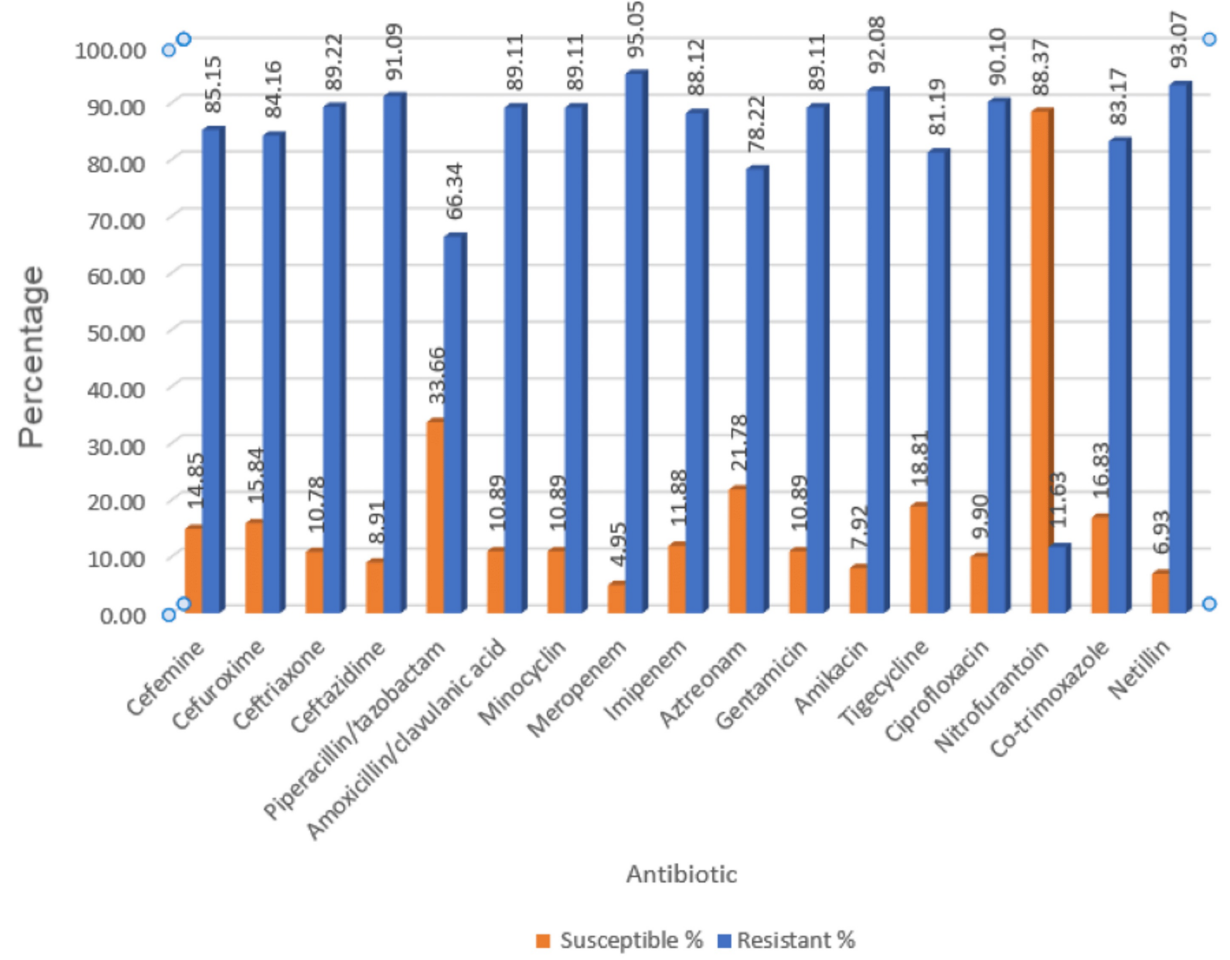
Antimicrobial susceptibility pattern of MDR Gram-negative bacilli. MDR: multidrug-resistant

The antibiotic susceptibility pattern demonstrated high resistance among the isolates to the most commonly used antimicrobials. The highest resistance was observed to meropenem (96, 95.05%), followed by netilmicin (94, 93.07%), amikacin (93, 92.08%), and ceftazidime (92, 91.09%). Cephalosporins and fluoroquinolones also demonstrated poor susceptibility. In contrast, nitrofurantoin showed low resistance (5, 11.63%) and high susceptibility (38, 88.37%) among urine isolates. Piperacillin/tazobactam was the second most frequently susceptible antimicrobial, with susceptibility observed in 34 isolates (33.66%), and it remained one of the more effective therapeutic options in this study. Overall, the findings indicate a high burden of multidrug-resistant organisms with limited treatment options (Figure 5).

## Discussion

Multidrug-resistant Gram-negative bacilli (MDR-GNB) represent a major challenge in intensive care units (ICUs). Critically ill patients are at increased risk of healthcare-associated infections due to prolonged hospital stays, the use of invasive devices such as urinary catheters, endotracheal tubes, and central venous lines, and frequent exposure to broad-spectrum antibiotics. These factors create favorable conditions for the emergence and spread of resistant organisms [12,13].

The present study evaluated the prevalence of MDR Gram-negative bacilli in ICU clinical isolates, identified predominant bacterial pathogens, and assessed associated specimen sources. In addition, antimicrobial susceptibility and resistance patterns were analyzed to determine the effectiveness of commonly used antibiotics. The findings provide valuable insights to guide empirical therapy, improve antimicrobial stewardship, and strengthen infection control strategies in ICU settings.

The results demonstrated that the prevalence of MDR-GNB infections varied across different age groups, with the highest prevalence observed in the 31-40 and 41-50 years age groups (26, 25.74%), followed by the 21-30 years age group (18, 17.82%). The lowest prevalence was noted in the 11-20 years and 71-80 years age groups (2, 1.98%). These findings are comparable to those reported by Çelik et al., who observed a higher proportion of MDR-GNB infections among patients aged 21-40 years (38.1%) [14].

Gender-wise distribution revealed male predominance, with 69 of 101 isolates (68.32%) obtained from male patients, compared with 32 (31.68%) from female patients. Similar findings were reported by Çelik et al., where male ICU patients constituted 64.6% of cases, suggesting a higher susceptibility among male patients to ICU-acquired MDR infections [14].

In the present study, *Pseudomonas aeruginosa* was the predominant MDR Gram-negative bacilli (31, 30.69%), followed by Klebsiella spp. (25, 24.75%), *Klebsiella pneumoniae* (18, 17.82%), *Escherichia coli *(12, 11.88%), and Acinetobacter spp. (11, 10.89%). Similar findings were reported by Bassetti et al. [9], Gagneja et al. [15], and Joseph and Boloor, who identified Pseudomonas, Klebsiella, and Acinetobacter species as predominant multidrug-resistant pathogens in ICU settings [16]. Although *Enterobacter cloacae *complexwas the least frequently isolated organism, accounting for 0.99% (1) isolate, it remains clinically significant because of its association with opportunistic and device-related infections [17].

Specimen-wise analysis revealed that urine was the most common source of MDR-GNB (43, 42.57%), followed by endotracheal tube (ETT) samples (22, 21.78%), pus/wound swabs (10, 9.90%), sputum and blood (9, 8.91% each), and body fluids (5, 4.95%). Minimal isolates were obtained from central venous pressure (CVP) tips, tracheostomy tubes, and Foley catheter tips (0.99% each). These findings are comparable to studies by Vincent et al. [3], Gong et al. [10], and Maina et al., which also reported urinary and respiratory samples as the predominant sources of MDR-GNB infections [13]. This may be attributed to the frequent use of urinary catheters and ventilatory support devices in ICU patients.

The antimicrobial susceptibility pattern demonstrated high resistance among MDR-GNB isolates to commonly used antibiotics. The highest resistance was observed to meropenem (96, 95.05%), followed by netilmicin (94, 93.07%), amikacin (93, 92.08%), and ceftazidime (92, 91.09%). Cephalosporins and fluoroquinolones also exhibited poor susceptibility. In contrast, nitrofurantoin demonstrated low resistance (5, 11.63%) and high susceptibility (38, 88.37%) among urinary isolates, indicating its effectiveness against urinary MDR-GNB isolates in the present study. Piperacillin/tazobactam was the second most frequently susceptible antimicrobial agent, with susceptibility observed in 34 isolates (33.66%), and remained one of the comparatively effective therapeutic options in this study.

These findings are consistent with previous reports by Gong et al. [10], Maina et al. [13], and Çelik et al., which documented high resistance rates among MDR Gram-negative bacilli in ICU settings [14]. The high resistance to carbapenems suggests the possible presence of carbapenemase-producing organisms, along with extended-spectrum β-lactamase (ESBL) production. The higher resistance observed to meropenem and imipenem compared to piperacillin-tazobactam may be attributed to prolonged and frequent empirical carbapenem use in ICU settings, resulting in selective antibiotic pressure and the emergence of carbapenem-resistant organisms. In addition, carbapenem resistance may occur through carbapenemase production, porin channel mutations, reduced membrane permeability, and efflux pump activity. In contrast, piperacillin-tazobactam may still retain activity against certain isolates lacking these specific resistance mechanisms, which may explain its comparatively better susceptibility pattern in the present study. Overall, MDR Gram-negative organisms were highly prevalent among ICU patients, resulting in limited therapeutic options. These findings highlight the urgent need for antibiotic susceptibility-guided therapy and the implementation of robust antimicrobial stewardship programs to control the spread of resistant pathogens and improve clinical outcomes.

Limitations

This study has certain limitations. It was conducted at a single institute in an intensive care setting, which may limit the generalizability of the findings. Molecular testing to identify specific resistance mechanisms was not performed, and long-term patient outcomes and treatment responses were not assessed. Despite these limitations, this study provides important insights into the prevalence, specimen sources, and antibiotic resistance patterns of multidrug-resistant Gram-negative bacilli in critically ill patients.

## Conclusions

This study highlights a high prevalence of multidrug-resistant Gram-negative bacilli (MDR-GNB) in ICU patients, with *Pseudomonas aeruginosa, *Klebsiella spp., Acinetobacter spp., and* Escherichia coli* identified as the most common pathogens. Infections were most frequent among middle-aged adults (31-50 years) and showed a male predominance. Urine and respiratory specimens were the primary sources, likely due to the use of urinary catheters and mechanical ventilation. The isolates demonstrated high levels of resistance to commonly used antibiotics, including carbapenems, aminoglycosides, cephalosporins, and fluoroquinolones, while nitrofurantoin remained relatively effective among urine isolates. Piperacillin/tazobactam was the second most effective antimicrobial agent observed in this study. These findings emphasize the importance of antibiotic susceptibility-guided therapy, strict infection control measures, and robust antimicrobial stewardship programs to reduce the burden of MDR infections in ICU settings.
